# Single nucleotide polymorphisms in *CETP*, *SLC46A1*, *SLC19A1*, *CD36*, *BCMO1*, *APOA5*, and *ABCA1* are significant predictors of plasma HDL in healthy adults

**DOI:** 10.1186/1476-511X-12-66

**Published:** 2013-05-08

**Authors:** Andrew J Clifford, Gonzalo Rincon, Janel E Owens, Juan F Medrano, Alanna J Moshfegh, David J Baer, Janet A Novotny

**Affiliations:** 1 Department of Nutrition, University of California, One Shields Avenue, Davis, CA 95616, USA; 2 Department of Animal Science, University of California, One Shields Avenue, Davis, CA 95616, USA; 3 Department of Chemistry and Biochemistry, University of Colorado Colorado Springs, 1420 Austin Bluffs Parkway, Colorado Springs, CO 80918, USA; 4 Food Surveys Research Group, US Department of Agriculture, Beltsville, MD 20705, USA; 5 US Department of Agriculture, Food Components and Health Laboratory, Beltsville, MD 20705, USA

**Keywords:** Single nucleotide polymorphism, HDL, Folate transporter, Cholesterol

## Abstract

**Background:**

In a marker-trait association study we estimated the statistical significance of 65 single nucleotide polymorphisms (SNP) in 23 candidate genes on HDL levels of two independent Caucasian populations. Each population consisted of men and women and their HDL levels were adjusted for gender and body weight. We used a linear regression model. Selected genes corresponded to folate metabolism, vitamins B-12, A, and E, and cholesterol pathways or lipid metabolism.

**Methods:**

Extracted DNA from both the Sacramento and Beltsville populations was analyzed using an allele discrimination assay with a MALDI-TOF mass spectrometry platform. The adjusted phenotype, *y*, was HDL levels adjusted for gender and body weight only statistical analyses were performed using the genotype association and regression modules from the SNP Variation Suite v7.

**Results:**

Statistically significant SNP (where *P* values were adjusted for false discovery rate) included: *CETP* (rs7499892 and rs5882); *SLC46A1* (rs37514694; rs739439); *SLC19A1* (rs3788199); *CD36* (rs3211956); *BCMO1* (rs6564851), *APOA5* (rs662799), and *ABCA1* (rs4149267). Many prior association trends of the SNP with HDL were replicated in our cross-validation study. Significantly, the association of SNP in folate transporters (*SLC46A1* rs37514694 and rs739439; *SLC19A1* rs3788199) with HDL was identified in our study.

**Conclusions:**

Given recent literature on the role of niacin in the biogenesis of HDL, focus on status and metabolism of B-vitamins and metabolites of eccentric cleavage of β-carotene with lipid metabolism is exciting for future study.

## Background

Understanding the effects of genetic, environmental, and especially of lipid levels on health status, is of wide and significant interest [1]. The relationships between persistent environmental pollutants and micronutrient levels are not well understood; though such speculation includes the role of peroxisome proliferator-activated receptor (PPAR), transcription factors related to lipid homeostasis, or changes in DNA methylation patterns [1]. In our recent work [2], we investigated 65 single nucleotide polymorphisms (SNP) in 23 candidate genes involved in folate metabolism (8 genes), vitamins B-12, A, and E metabolism (5 genes), and cholesterol pathways or lipid metabolism (10 genes) in a homocysteine/red blood cell folate marker trait association study. Interestingly, a few SNP associated with diabetes mellitus (DM), cardiovascular disease (CVD) and maintenance of the cholesterol pathway or lipid metabolism were identified: serine palmitoyltransferase (*SPTLC1* rs11790991), cholesteryl ester transfer protein (*CETP* rs5882) and scavenger receptor class B type 1 (*SCARB1* rs838892). SNP associated with transfer of antioxidant vitamins, including rs2118981 in the cellular retinol binding protein II gene (*CRBP2*), which is important for vitamin A and retinoid transfer, and rs838892 *SCARB1* (for tocopherols and tocotrienols), were also statistically significant predictors in the final model. Significantly, SNP in betaine-homocysteine methyltransferase (*BHMT* rs3733890) and methylene tetrahydrofolate reductase (*MTHFR* rs1801131), both of which are involved in one-carbon metabolism, were also included in the final model that was previously described [2].

Given the establishment of these associations in our previous work [2], further investigation of the relation of folate, homocysteine (Hcy), and changes in plasma lipid profiles was of significant research interest. High levels of Hcy and changes in plasma lipids are independent risk factors for development of CVD, and there may be a connection between Hcy metabolism and lipid metabolism [3]. Low levels of dietary folate, an important cofactor in the metabolism of Hcy, led to increased levels of serum and liver cholesterol in wild-type mice [4]. As well, cholesterol metabolism may contribute to beneficial effects of dietary folate supplementation [4]. Possible connections, not yet fully supported by experimental data, between folate levels and cholesterol biosynthesis include endoplasmic reticulum stress, which activates cholesterol biosynthesis genes, or through metabolic processes involving choline. Choline provides methyl groups for Hcy metabolism and is a precursor for choline phospholipids, which are required for lipoprotein secretion [4]. Additionally, serum lipid levels are risk factors for a number of adverse health outcomes, including coronary heart disease (CHD), atherosclerosis, type 2 DM, stroke, and metabolic syndrome [1]. Lipid levels of interest include triglycerides, low-density lipoprotein (LDL), and high-density lipoprotein (HDL), especially as recent work has indicated that HDL may not always be protective against atherosclerosis [5,6]. Particularly, the functionality and quality of HDL [7] in response to vascular inflammation and oxidative stress [5] is an interesting area of further study. HDL is required in the reverse cholesterol transport (RCT) mechanism where excess cholesterol is transferred from peripheral cells to the liver for intestinal excretion [8]. Additionally, HDL has anti-inflammatory and antioxidant effects and improves endothelial function [8].

In this study we selected 65 SNP in 23 candidate genes to perform a marker trait association study with plasma HDL adjusted for gender and body weight in Caucasian male and female study participants from two independent populations (Sacramento, California, and Beltsville, Maryland). Selected SNP corresponded to 8 genes associated with folate metabolism, 5 genes associated with vitamin B-12, vitamin A and vitamin E metabolism, and 10 genes associated with cholesterol pathways or lipid metabolism. The overall objective of this study was to determine if any of these SNP in the 23 candidate genes had a statistically significant association with HDL.

## Results

### Significant SNP predictors

Tables 1 and 2 include significant SNP predictors on HDL concentrations (mg/dL) that were validated in both the Sacramento and Beltsville populations, respectively. Each table lists the gene with corresponding statistically significant SNP predictor (with associated *P*-value) and the square of the correlation coefficient (R^2^) indicating the proportion of response variation explained by the regressors in the model. The ASE in Tables 1 and 2 represents the allele substitution effect, which is the slope derived from the regression, and where a positive ASE indicated a positive association with the adjusted HDL and conversely, a negative ASE indicated a negative association. The false discovery rate (FDR) was corrected for any chance findings. The minor allele frequency (MAF) is the frequency at which the less common allele occurs in the population indicating also the frequency of the allele causing the effect.

**Table 1:** Sacramento population

**Gene**	**SNP predictor**	***P*-value**	**R**^**2**^	**ASE**	**FDR**	**MAF**
*CETP*	rs7499892	0.000437	0.098	−2.309	0.0015	0.15
*CETP*	rs5882	2.16E-05	0.205	−10.250	0.0004	0.05
*SLC46A1*	rs35714695	2.62E-06	0.160	−6.644	0.0002	0.15
*SLC46A1*	rs739439	6.03E-06	0.207	−6.713	0.0002	0.17
*SLC19A1*	rs3788199	0.000209	0.226	2.828	0.0012	0.45
*CD36*	rs3211956	9.39E-08	0.183	4.372	1.14E-06	0.07
*BCMO1*	rs6564851	0.000456	0.114	0.586	0.0014	0.49
*APOA5*	rs662799	0.000491	0.148	−4.523	0.0013	0.05
*ABCA1*	rs4149267	4.59E-08	0.195	−3.236	9.76E-07	0.37

Significant SNP predictors on HDL concentrations (mg/dL) in the Sacramento population (*n* = 249).

**Table 2:** Beltsville population

**Gene**	**SNP predictor**	***P*-Value**	**R**^**2**^	**ASE**	**FDR**	**MAF**
*CETP*	rs7499892	3.10E-05	0.097	−2.088	0.0002	0.16
*CETP*	rs5882	9.57E-05	0.086	−2.145	0.0002	0.04
*SLC46A1*	rs35714695	6.21E-05	0.091	1.606	0.0002	0.17
*SLC46A1*	rs739439	8.33E-05	0.091	1.323	0.0002	0.18
*SLC19A1*	rs3788199	0.001037	0.085	1.796	0.0100	0.41
*CD36*	rs3211956	7.93E-05	0.089	1.774	0.0002	0.07
*BCMO1*	rs6564851	7.90E-05	0.088	1.015	0.0002	0.49
*APOA5*	rs662799	6.72E-05	0.091	−0.471	0.0002	0.06
*ABCA1*	rs4149267	0.00143	0.071	−2.070	0.0078	0.38

Significant SNP predictors on HDL concentrations (mg/dL) in the Beltsville population (*n* = 532).

Statistically significant SNP predictors in the Sacramento and Beltsville populations were similar between both groups though there were some differences in the directionality and magnitude of the association between SNP predictor and HDL. SNP of genes that were statistically significant included: 1) cholesteryl ester transfer protein (*CETP*; rs7499892 and rs5882); 2) proton-coupled folate transporter (*SLC46A1*; rs35714695 and rs739439); 3) reduced folate carrier (*SLC19A1*; rs3788199); 4) thrombospondin receptor (*CD36*; rs3211956); 5) beta-carotene monooxygenase 1 (*BCMO1*; rs6564851); 6) apolipoproteinA-V (*APOA5*; rs662799); and 7) ATP-binding cassette transporter member 1 (*ABCA1*; rs4149267).

The two SNP included in this study for *CETP* (rs7499892 and rs5882) with MAF of ~0.16 and MAF of ~0.04 for the rare allele, respectively, were statistically significantly negatively associated with HDL-C in both the Sacramento and Beltsville populations (Tables 1 and 2) as indicated by the ASE (slope of the regression). It was determined that the rs3788199 SNP in *SLC19A1* was positively correlated with HDL levels (ASE = 2.828 for the Sacramento population, Table 1; ASE = 1.796 for the Beltsville population, Table 2). The SNP rs35714695 and rs739439 of *SLC46A1* were both negatively associated with HDL levels in the Sacramento population though the Beltsville population had a positive association with HDL levels, which indicates that the allele effects are opposite. The directionality of this association difference is one area of future study. In the present study, a very large positive association between HDL levels and the presence of rs3211956 of *CD36* was also identified for both study populations (Table 1 and Table 2). In the Sacramento population, the ASE was 4.372, with similar positive association (but not as large) found in the Beltsville population (ASE = 1.774). The results of the present study support a past finding: in both populations (Sacramento and Beltsville), a positive association, as indicated by the positive ASE values (Table 1, ASE = 0.586 and Table 2, ASE = 1.015), was established for the rs6564851 SNP of *BCMO1* and plasma HDL levels. For *APOA5*, the rs662799 SNP was statistically significantly negatively associated with HDL. The ASE was −4.523 for the Sacramento population (Table 1) and −0.471 for the Beltsville population (Table 2), indicating that there was a negative association of the presence of this SNP with measured HDL concentrations. Finally, the SNP rs4149267 of *ABCA1* was associated with HDL-C in both Caucasian populations with similar ASE of −3.236 in the Sacramento population (Table 1) and −2.070 in the Beltsville population (Table 2).

## Discussion

### CETP

*CETP* encodes cholesteryl ester transfer protein, which exchanges the triglycerides from VLDL and LDL particles for cholesterol esters from HDL. CETP also selectively enhances liver HDL cholesterol ester uptake [9]. Inhibiting CETP with various pharmacologic agents (torcetrapib, dalcetrapib, anacetrapib, evacetrapib) has been an attractive means to minimize risk for adverse cardiovascular events because of a potential rise in HDL-C and fall in LDL-C [8,10]. Understanding the molecular basis for cholesteryl ester transport by CETP is of research interest to support the development of other CETP inhibitors [11]. In a previous genome wide association study (GWAS), the SNP rs708272 (or *Taq*IB SNP) of *CETP* had the strongest association with HDL-C of all 948 SNPs genotyped in 122 genes though this SNP was not included in our study. As well, this SNP had a strong association with apolipoproteinA-1 (apoA1) [12]. In a population of Chinese Bama Zhuang individuals, the rs708272 SNP was associated with higher HDL-C levels, but the outcome of this SNP on longevity was less clear [13]. A recent meta-analysis of 46 lipid GWAS, six HDL-C loci were identified with at least a second independent association with HDL-C including *LPL, ABCA1, APOA1/A4/A5/C3, ZNF664, LIPC,* and *CETP*[14]. Both *ABCA1* and *CETP* were validated in our study. Tietjen and colleagues showed that rare coding and splicing mutations on *CETP* were enriched in persons with hyperalphalipoproteinemia and segregated with elevated HDL-C in families [15].

In our prior study, rs5882 of *CETP* was statistically significantly associated with Hcy normalized by red blood cell folate concentrations [2]. Two of the four SNP included in this study for *CETP* (rs7499892 and rs5882) were statistically significantly negatively associated with HDL-C in both the Sacramento and Beltsville populations (Tables 1 and 2). The rs5882 SNP has been associated with lower CETP serum concentrations and activity, increased HDL cholesterol levels, and increased lipoprotein sizes, all factors which have been associated with a lower CVD risk [16]. In a recent study in Tunisian population, there was no statistically significant association of the rs5882 SNP with lipoprotein metabolism or atherogenicity [17].

### SLC46A1 and SLC19A1

The proton-coupled folate transporter (PCFT, gene symbol *SLC46A1*) mediates intestinal folate absorption and folate transport across the choroid plexus. PCFT has an optimal pH transport of 5.0 – 5.5 (jejunum and duodenum), but the role of this transporter in other tissues at normal physiological pH is less clear [18]. Homozygous mutations in *SLC46A1* have been associated with a rare disorder, hereditary folate malabsorption [18]. Solute carrier family 19 (folate transporter) member 1, also known as the reduced folate carrier (RFC; gene symbol *SLC19A1*), is involved in the regulation of intracellular concentrations of folate. Higher serum folate concentrations have been associated with lower levels of LDL-C, lower LDL-C/HDL-C ratio, and higher HDL-C. These associations were independent of gender or age, though influences of medications, diseases, physical exercise, diet, or BMI were not accounted for in that study. Interestingly, vitamin B12 was not associated with the lipoprotein profile in that reported study [19]. Cholesterol may be important for facilitating the import of folate by clustering membrane-bound folate receptors in the cell membrane [19]. Use of clustering membrane-bound folate receptors was favoured when folate status was low [20]. Folate status is inversely associated with obesity [21], likely due to increased activity of COMT (catechol *O-*methyltransferase), which uses folate for methyl transfer for metabolism of catechol estrogen produced by adipose tissue. Both obesity [21] and low folate status [19] have been associated with reduced HDL cholesterol levels. However, in a recent study examining folate placental transport in obese women, it was determined that while protein expression of folate receptor-α and RFC were altered (PCFT was unchanged), the activities of the transporters was unaltered in obesity and fetal folate serum concentration were not adversely affected [22]. In the present study, it was determined that the rs3788199 SNP in *SLC19A1* was positively correlated with HDL levels (ASE = 2.828 for the Sacramento population, Table 1; ASE = 1.796 for the Beltsville population, Table 2), which compares well to the earlier retrospective database study previously described [19]. Surprisingly, it was also determined that the SNP rs35714695 and rs739439 of *SLC46A1* were both negatively associated with HDL levels in the Sacramento population whereas the Beltsville population had a positive association with HDL levels, which indicates that the allele effects are opposite. To our knowledge, there are no studies providing information on association studies of SNP in *SLC46A1* with HDL cholesterol levels. Given the differences in association between the two populations in this study, investigation of SNP in *SLC46A1* with HDL is of further research interest.

A 2010 study examined seven SNP variants in genes involved in Hcy metabolism on the interaction with plasma lipid profile. In that study, *SLC19A1* (SNP rs1051266) was not found to have a statistically significant association with blood lipid profiles analysed (fasting lipoprotein, total cholesterol, LDL, HDL, and triglyceride levels) [3]. Additional findings from that study implicated SNP in the genes for transcobalamin II (rs1801198) and *MTHFR* (rs1801133) were associated with blood lipid profiles. The G allele of rs1801198 was correlated with higher levels of LDL in plasma, lower HDL, higher triglyceride levels, and higher total cholesterol levels [3]. However, in our study, neither rs1801198 (transcobalamin II) nor rs1801133 (*MTHFR*) were statistically significantly associated with levels of HDL in plasma.

### CD36

CD36 (thrombospondin receptor), or glycoprotein IIIb/platelet glycoprotein IV is a mediator of platelet adhesion to collagen. The investigation of SNP of *CD36* on HDL is not well established, likely because participants in many studies deal with chronic diseases, including DM, CHD, or metabolic syndrome or risk factors for chronic diseases [23-26]. Therefore, *CD36* warrants further investigation, with careful statistical control of potentially confounding variables, including environmental factors, dietary factors, and other genotypes. In our study, we enrolled healthy participants as indicated by low use of statins (less than ~7% for overall study population) and healthy BMI (mean ± standard deviation for Sacramento was 24.8 ± 4.2 and 26.6 ± 4.6 for Beltsville). We found that there was a very large positive association between HDL levels and the presence of SNP rs3211956 (Tables 1 and 2). In the Sacramento population the ASE was 4.72, with similar positive association (but not as large) found in the Beltsville population (ASE=1.774).

### BCMO1

Beta-carotene monooxygenase 1 (BCMO1) catalyzes the first step in the central cleavage and conversion of dietary provitamin carotenoids to vitamin A (retinal) in the small intestine [27,28]. Vitamin A is necessary for immune response, vision, embryonic development, cell differentiation, and membrane and skin protection [27]. The statistically significant SNP identified in the present study, rs6564851, is 7.7 kb 5’ upstream from the *BCMO1* gene. This particular SNP has been associated with a 48% reduced catalytic activity of converting β-carotene into vitamin A in female participants in a recent study [29]. Other SNP in *BCMO1* have been associated with plasma levels of various carotenoids, including β-carotene, lutein, α-carotene, zeaxanthin, and lycopene [27]; and the G allele of the rs6564851 may explain some of the variance in plasma levels of these provitamin carotenoids. The rs6564851 SNP may be particularly important for individuals at risk for vitamin A deficiency [27] owing to reduced catalytic activity of BCMO1 [29]. Recently, the rs6564851 SNP had the strongest association (when compared to other SNP: rs11645428, rs6420424, and rs8044334) with fasting β-carotene concentrations in plasma [29]. Higher levels of carotenoids (isomers of β-carotene, lycopene, and β-cryptoxanthin) have been associated with higher levels of HDL and LDL in a recent study involving NHANES participants [1]. In a recent review, the important physiological effects of eccentric cleavage products of beta-carotene were discussed. Considering the effects of *BCMO1* SNP as well, there could be some very different physiological effects from beta-carotene consumption owing to genetic influences, oxidative stress, and presence of various beta-carotene metabolites [28]. Interesting recent work has focused on the retinoid receptor antagonist activity of products resulting from β-carotene eccentric cleavage, the β-apocarotenoids [30,31]. The biological activities and effects of these β-carotene metabolites may be important to consider in evaluating oxidative stress and adverse health outcomes of CVD or cancer [30]. It has been established that there is no association between the *BCMO1* SNP rs6564851 and risk of developing type 2 DM [32].

### APOA5

ApolipoproteinA-V is a protein component of HDL. In this study, the rs662799 SNP of *APOA5* was identified as being a significant predictor. The ASE was −4.523 for the Sacramento population and −0.471 for the Beltsville population, indicating that there was a negative association of the presence of this SNP with measured HDL concentrations. In a recent study [33], the rs662799 SNP was the only SNP (of thirteen evaluated in the *APOA1/C3/A4/A5* gene cluster) to be associated with three lipid traits: triglycerides, HDL-C, and LDL-C levels. In this past study, the MAF was statistically significantly associated with familial combined hyperlipidaemia, though the functional effect of this rs662799 SNP may not be well understood [33]. In another recent study, rs662799 was statistically significantly associated with plasma triglycerides in both women and men of the study population and statistically significantly associated with total cholesterol and LDL-C levels in men only. However, the authors concluded that haplotypes for five SNP in the apolipoprotein A1/C3/A5 cluster could explain more serum lipid variation than any one SNP alone, especially for HDL-C [34]. The presence of the rs662799 SNP was statistically significantly associated with lower levels of total cholesterol, triglycerides, and LDL-C in a group of Hei Yi Zhuang Chinese (though this same trend was not observed in Han Chinese), indicating that there may be other gene-gene or gene-environment interactions [35].

### ABCA1

ABCA1 (ATP-binding cassette transporter member 1) plays an important role in cellular cholesterol and phospholipid homeostasis in multiple cell types [36,37] and is involved in RCT [38]. ABCA1 mediated efflux of cholesterol and phospholipids leads to the formation of nascent HDL via apoA1 [39,40]; and mutations that disrupt normal ABCA1 function result in little or no circulating HDL [41]. ABC transporter G1 (ABCG1) promotes cholesterol efflux from macrophages to HDL to form mature HDL particles [40], and hence works in a sequential manner with ABCA1 [42]. All trans-retinoic acid has been shown to increase apoA1/HDL-mediated cholesterol efflux from macrophages by increasing ABCA1 and ABCG1 by regulating promoter activity via liver X receptor-responsive element mechanism [42]. Wiersma and colleagues [43] also showed that *ABCG1* knock-out mice exhibit decreased HDL-C when consuming a high fat diet. In this study, they also demonstrated that ABCG1 mediated cholesterol efflux to HDL [43]. Functional mutations in ABCA1 cause Tangier disease, which is characterized by very low levels of plasma HDL apoA1 [44,45]. In a recent study investigating exome sequencing, functional rare variants in *ABCA1* and *LPL* (gene for lipoprotein lipase) were identified and explained a major portion of the HDL-C variance in the population enrolled in the study [41].

Previous studies have found associations between certain SNP in *ABCA1* and HDL concentrations [46-48]. Recent GWAS and meta-analysis studies showed that SNP in *ABCA1* were significantly associated with HDL-C [14,49]. In our study, the SNP rs4149267 of *ABCA1* was associated with HDL-C in both Caucasian populations with similar ASE of −3.236 in the Sacramento population and −2.070 in the Beltsville population.

It would be significant to understand the effects of apolipoprotein E (apoE), which plays an important role in lipoprotein metabolism and atherosclerosis. ApoE has been shown to promote selective uptake of HDL-C owing to increased ABCA1-mediated cholesterol efflux to plasma [50]. This present work identifies significant SNP in *ABCA1* and hence this relation with *ABCA1* SNP and apoE facilitated HDL-C transport is of future research interest.

## Conclusions

The strength of our study is the cross-validation between two independent populations (one on the West coast, from Sacramento, CA, and one from the East coast, from the Washington, D.C. area) with similar SNP associations identified in both populations. It would be of significant research interest to focus on the relation of B vitamins on HDL status. In this work, we have identified SNP in two folate transporters (*SLC46A1* rs35714695 and rs739439; *SLC19A1* rs3788199) having statistically significant ASE in relation to HDL status in both study populations. Cholesterol may be important for facilitating the import of folate across the cell membrane and higher serum folate concentrations have been associated with lower levels of LDL-C and higher levels of HDL-C [19]. Past work by Kitami et al. focused on the importance of the homeostatic role of cholesterol metabolism on folate retention in mouse strains, so there has been an established relationship between cholesterol and folate in the mouse [4]. Recent work by Zhang et al. identified the role of niacin (also known as vitamin B3) on early hepatic HDL formation through transcription of *ABCA1*. In that study, apoA1 lipidation and formation of nascent HDL was mediated and stabilized by niacin, which may prevent premature HDL catabolism [51].

Finally, the identification of the positive association of the *BCMO1* SNP rs6564851 with HDL levels was of significance. This SNP has a high MAF in the two independent study populations of this work (Tables 1 and 2). Additionally, the presence of this SNP has been associated with a 48% reduction in activity of converting β-carotene into vitamin A through central cleavage, resulting in higher circulating levels of plasma carotenoids [29]. These higher levels of carotenoids may be associated with higher levels of HDL and LDL [1]. The biological effects of the eccentric cleavage products of β-carotene (the apo-β-carotenoids), especially on lipid metabolism and oxidative stress, are an exciting area of future study.

## Methods

### Study populations

#### Sacramento population

The Institutional Review Board of the University of California, Davis, approved the study, which was conducted according to Good Clinical Practice guidelines and the Declaration of Helsinki, version 1989. Written informed consent was obtained from each participant before enrollment in the study. Women and men ranging in age from 18 to 67 years were recruited by posted, published, and mailed advertisements in the California counties of Sacramento, San Joaquin, Solano, and Yolo from May 2004 through August 2005. Persons were excluded for any history of a serious medical condition, for using medications that could interfere with folate metabolism (including phenytoin, primidone, fosphenytoin, carbamazepine, phenobarbital, ranitidine, trimethoprim, metformin, sulfasalazine, triamterene or methotrexate, beta blockers, and angiotensin inhibitors), for using tobacco, or for heavy consumption of alcohol (≥ 3 drinks per day). Responders to advertisements enrolled in a single clinic visit at the Ragle Human Nutrition Research Center at the University of California, Davis. Before the clinic visit, participants in the study received via US mail a packet containing information about the study, consent forms, instructions to fast for 8 – 10 h prior to the clinic visit, and two dietary intake instruments to assess folate intake from the diet and supplements. Folate intake assessed by the Block Dietary Folate Equivalents (DFE) Screener is available through Supporting Information (Additional file 1: Figure S1). At the time of the scheduled visit, participants were interviewed about general medical, personal, and family histories. Gender, age, and BMI (calculated from height and weight measurements) were also recorded. Fasting whole blood samples were then drawn as described below. A $15 gift certificate to a local supermarket or department store was given to each person at the end of the clinic visit. Two hundred forty-nine individuals were enrolled in the Sacramento population.

#### Beltsville population

The Johns Hopkins University Bloomberg School of Public Health Committee on Human Research approved the study procedures and protocol, which was in accord with Good Clinical Practice guidelines and the Declaration of Helsinki, version 1989. The population consisted of 510 adults (257 females, 253 males) aged 30 – 69 years residing in the greater Washington, D.C. area. Participants were recruited to participate in a study of dietary recall between July 2002 and June 2004 [52] and were included in the present study as an adjunct to the previous study. Potential participants were recruited through email, advertisements in local newspapers, and announcements on USDA ARS websites. Participants attended an informational meeting concerning study procedures before completing a health history questionnaire. The questionnaire covered general medical, personal, and family histories. Folate intake assessed by the Block Dietary Folate Equivalents (DFE) Screener is available through Supporting Information (Additional file 1: Figure S1). A medical screening evaluation included measurement of height, weight, blood pressure, and laboratory analysis of fasting blood and urine. Age, BMI (calculated from height and weight measurements) and gender were also recorded. The medical history and laboratory results were reviewed by study investigators and a cooperating physician to confirm good health of the participants and that there was no evidence of underlying disease, untreated thyroid disorders, gastrointestinal disease, malabsorption syndromes, history of eating disorders, cancer, or DM. All participants were weight stable, were not actively pursuing a weight-loss regimen, were not taking medications known to affect food intake or appetite, and were not taking diuretics or other medications that may affect water balance. Pregnant and lactating women were excluded from the study. Participants were compensated according to the requirements of the main study [52].

### Blood analysis and genotyping

#### *Sacramento population*

Fasting whole blood was collected in triplicate. It was collected 1) into spray-dried K_2_EDTA tubes for assessment of complete blood count, red blood cell (RBC) folate, plasma vitamin B6, and plasma Hcy; 2) into serum separator tubes for the assessment of serum vitamin B12, serum folate, and lipid panel; and 3) into 8.5-mL whole blood DNA tubes for the assessment of genomic DNA. The preparation and analysis of the collected blood samples has been previously described [2]. The results of RBC folate concentrations and plasma vitamin B12 levels are included in the online *Supporting Information* (Additional file 1: Figures S2 and S3). Briefly and specifically to this study, the lipid panel was completed using the Beckman LXI and LX20 Pro (Beckman Instruments, Brea, CA) at the Department of Pathology laboratory of the University of California, Davis. Results of serum triglycerides, total cholesterol, HDL-C, and LDL-C are provided in the online *Supporting Information* (Additional file 1: Figures S4 S5, S6, S7). Additionally, genomic DNA was extracted from whole blood with the use of a PAXgene Blood DNA kit (PreAnalytix; Qiagen, Inc., Valencia, CA).

#### Beltsville population

Blood was collected into serum separator tubes after a 12 h fast. Blood samples were allowed to sit for 30 min before centrifugation at 2000 × *g* for 10 min at 4°C then aliquotted and stored at −80°C until analysis. Plasma-deplete samples were analysed for folate concentration as described previously [2]. The results of RBC folate concentrations and plasma vitamin B12 levels are included in the online *Supporting Information* (Additional file 1: Figures S2 and S3). Thawed samples were analyzed in duplicate for HDL cholesterol on a Dade Behring Dimension xPand clinical chemistry analyzer (Siemens, Erlangen, Germany). Results of serum triglycerides, total cholesterol, HDL-C, and LDL-C are provided in the online *Supporting Information* (Additional file 1: Figures S4, S5, S6, S7). High quality DNA was extracted from white blood cells using the Gentra PureGene Blood DNA Purification Kit (Qiagen, Inc.) as was previously described [2] prior to SNP analysis.

### Genotyping of SNP

Extracted DNA from both the Sacramento and Beltsville populations was analyzed using an allele discrimination assay with a MALDI-TOF mass spectrometry platform (Sequenom MassARRAY®) (Neogen/GeneSeek Inc., Lincoln, NE). A total of 65 SNP in 23 genes were analysed. Candidate gene selection was performed based upon a literature search of pathways involving folate, lipids, vitamins A, E, and B12 metabolism. Specific SNP in relevant genes were obtained from dbSNP [53] and Ensembl [54] databases (Table 3).

**Table 3:** SNP included in analyses

**Name**	**Gene, function**	**Chromosome**	**dbSNP identifier**	**n**
Apolipoprotein A1	*APOA1*, protein component of HDL, promotes cholesterol efflux from tissues to liver	11	rs2727784	1
Apolipoprotein A5	*APOA5*, protein component of HDL, regulates plasma triglycerides	11	rs3135506, rs662799, rs12272004	3
ATP-binding cassette, subfamily A, member 1	*ABCA1,* transport AAs, sugars, vitamins, cholesterol, pt-choline to lipid acceptor apoA1	9	rs2230806, rs2230808, rs4149267, rs4149327	4
β-Carotene 15,15’-monooxygenase 1	*BCMO1,* cleave β-carotene	16	rs12934922, rs6564851, rs7501331	3
β-Carotene oxygenase 2	*BCMO2*, cleave β-carotene	11	rs11214139, rs2250417, rs35361223	3
Betaine-homocysteine *S*-methyl transferase	*BHMT,* methylate Hcy	5	rs3733890	1
Cholesteryl ester transfer protein, plasma	*CETP,* transfer cholesterol	16	rs12708980, rs5880, rs5882, rs7205804, rs7499892	5
Cystathionine β-synthase	*CBS,* synthesize cystathionine	21	rs5742905	1
Cytochrome P450, family 4, subfamily F, polypeptide 2	*CYP4F2*, involved in cholesterol synthesis	19	rs3093156, rs2108622, rs3093168, rs3093194	4
Folate hydrolate (prostate specific membrane antigen) 1	*FOLH1 (GCPII), hydrolyze folate*	11	rs61886492	1
Methionine synthase reductase	*MTRR,* remethylate Hcy	5	rs1801394	1
Methylenetetrahydro-folate reductase	*MTHFR,* distribute one carbon units	1	rs1801131, rs1801133	2
5-Methyltetra-hydrofolate-homocysteine methyltransferase	*MTR,* remethylate Hcy	1	rs1805087	1
Microsomal triglyceride transfer protein	*MTTP*, lipoprotein assembly	4	rs10516445, rs1057613, rs3828542, rs881980	4
Niemann-Pick disease, type C1, gene-like 1	*NPC1L1*, absorption of intestinal cholesterol and α-tocopherol transport	7	rs11763759, rs217420, rs217430, rs217432	4
Retinol binding protein 1, cellular	*CRBP2 (RBP2),*	3	rs2118981, rs35674260, rs3772875	3
Scavenger receptor class B, member 1	*SCARB1,* intestinal absorption of carotenoids	12	rs10773105, rs12582221, rs7306660, rs7967521, rs838892	5
Serine palmitoyltransferase, long chain base subunit 1	*SPTLC1*, sphingolipid biosynthesis	9	rs11790991, rs2297568, rs7858659	3
Solute carrier family 19 (folate transporter), member 1	*SLC19A1,* reduced folate carrier	21	rs13050920, rs3788205, rs3788199	3
Solute carrier family 46 (folate transporter), member 1	*SLC46A1,* proton coupled folate transporter (PCFT)	17	rs11080058, rs1128162, rs17719944, rs35714695, rs739439	5
CD36 Thrombospondin receptor	*CD36*, platelet surface glycoprotein, binds to oxidized LDL	7	rs1358337, rs3211834, rs3211931, rs3211956	4
α-Tocopherol transfer protein	*α-TTP, transport α-tocopherol*	8	rs4501570, rs4587328, rs4606052	3
Transcobalamin II	*TCII, transport vitamin B12*	22	rs1801198	1
Total SNP included in analysis				65

Information regarding the 65 SNP included in the analysis. In the last column, *n* denotes the number of SNP per gene.

### Data processing and statistical analysis

#### Association analysis

Marker-trait association analysis was performed using a linear regression test under an additive model assumption in Caucasian participants from both study populations only. The adjusted phenotype, *y*, was HDL levels adjusted for gender and body weight only (see discussion in *Statistical significance of fixed effects*, below). Statistical analyses were performed using the genotype association and regression modules from the SNP Variation Suite (SVS) version 7 (Golden Helix Inc., Bozeman, MT). In brief, the adjusted phenotype, *y*, was fit to every encoded genotype under an additive model assumption, *x*, and was represented with the following equation (1):

(1) $$y=b_{1}x+b_{0}+\epsilon$$

Where *y* was the adjusted phenotype (HDL adjusted for gender and weight), *b*_*1*_*x + b*_*0*_ represented the model, and the error term, *ϵ*, expressed the random residual effect.

#### Statistical significance of fixed effects

Participant data (gender, age, height, weight, and calculated BMI) were tested to adjust phenotypes for systematic effects using a full (including covariates) versus reduced model regression equation. The regression sums of squares were calculated both for a reduced and for the full model. An F test was then performed to find the significance of the full versus the reduced model. A *P*-value threshold of 0.01 was used to establish significant associations. Gender and body weight effects were statistically significant; therefore, adjusted phenotypes were obtained for all samples.

The linear regression was also performed including SNP interactions using the SVS version 7 regression module from Golden Helix. FDR was controlled according to a previous method [55] and a cutoff for a significant association value was set at FDR q value < 0.01.

## Abbreviations

SNP: Single nucleotide polymorphism(s); HDL and HDL-C: High-density lipoprotein high-density lipoprotein cholesterol; CETP: Gene symbol for cholesteryl ester transfer protein; SLC46A1: Gene symbol for solute carrier family 46 (folate transporter), member 1 or proton-coupled folate transporter; SLC19A1: Gene symbol for solute carrier family 19 (folate transporter), member 1, or reduced folate carrier; CD36: Gene symbol for thrombosponding receptor or glycoprotein IIIb/platelet glycoprotein IV; BCMO1: Gene symbol for beta-carotene monooxygenase 1; APOA5: Gene symbol for apolipoproteinA-V; ABCA1: Gene symbol for ATP-binding cassette transporter member 1; PPAR: Peroxisome proliferator-activated receptor; DM: Diabetes mellitus; CVD: Cardiovascular disease; SPTLC1: Gene symbol for serine palmitoyltransferase; SCARB1: Gene symbol for scavenger receptor class B type 1; CRBP2: Gene symbol for cellular retinol binding protein; BHMT: Gene symbol for betaine-homocysteine methyltransferase; MTHFR: Gene symbol for methylene tetrahydrofolate reductase; Hcy: Homocysteine; CHD: Coronary heart disease; LDL and LDL-C: Low-density lipoprotein and low-density lipoprotein cholesterol; RCT: Reverse cholesterol transport; ASE: Allele substitution effect; FDR: False discovery rate; MAF: Minor allele frequency; VLDL: Very low-density lipoproteins; CETP: Cholesteryl ester transfer protein; GWAS: Genome-wide association study; apolA1: Apolipoproteina-1; PCFT: Proton-coupled folate transporter; RFC: Reduced folate carrier; COMT: Catechol *O*-methyltransferase; CD36: Thrombosponding receptor or glycoprotein IIIb/platelet glycoprotein IV; BMI: Body mass index; BCMO1: Beta-carotene monooxygenase 1; NHANES: National Health and Nutrition Examination Survey; APOA5: Apolipoproteina-V; ABCA1: ATP-binding cassette transporter member 1; ABCG1: ATP-binding cassette transporter G1; LPL: Gene symbol for lipoprotein lipase; apoE: Apolipoprotein E; K2EDTA: Potassium ethylenediaminetetraacetic acid; RBC: Red blood cell; MALDI-TOF: Matrix-assisted laser desorption ionization time-of-flight; DFE: Dietary Folate Equivalents.

## Competing interests

The authors declare that they have no competing interests.

## Authors' contributions

GR, JFM, and AJC designed the research; GR and JEO conducted the research; AJM, DJB, and JAN provided essential materials and data; GR performed statistical analyses and wrote the statistical methods section; JEO, GR, and AJC wrote the manuscript; DJB and JAN participated in manuscript preparation; and AJC had final responsibility for the content. All authors read and approved the final manuscript.

## Supplementary Material

Additional file 1: Figure S1 Diet Folate Equivalent (DFE) histogram of Sacramento (*n* = 248) and Beltsville (*n* = 505) study participants. **Figure S2:** Red Blood Cell (RBC) folate concentrations histogram of Sacramento (*n* = 248) and Beltsville (*n* = 509) study participants. **Figure S3:** Comparison of plasma vitamin B12 concentrations (pg/mL) in the Sacramento and Beltsville populations. **Figure S4:** Comparison of serum triglyceride levels in the Sacramento (*n* = 248) and Beltsville (*n* = 505) study participants. **Figure S5:** Comparison of total cholesterol (mg/dL) in the two study populations. **Figure S6:** Comparison of HDL cholesterol (mg/dL) in the two study populations. **Figure S7:** Comparison of LDL cholesterol (mg/dL) in the two study populations. Click here for file
