# Supplementary material for: Single nucleotide polymorphisms in CETP, SLC46A1, SLC19A1, CD36, BCMO1, APOA5, and ABCA1 are significant predictors of plasma HDL in healthy adults
Source: Lipids Health Dis. 2013 May 8;12:66. doi: 10.1186/1476-511X-12-66 (PMC3653731; doi:10.1186/1476-511X-12-66)
Supplement: Additional file 1: Figure S1 — Diet Folate Equivalent (DFE) histogram of Sacramento (n = 248) and Beltsville (n = 505) study participants. Figure S2: Red Blood Cell (RBC) folate concentrations histogram of Sacramento (n = 248) and Beltsville (n = 509) study participants. Figure S3: Comparison of plasma vitamin B12 concentrations (pg/mL) in the Sacramento and Beltsville populations. Figure S4: Comparison of serum triglyceride levels in the Sacramento (n = 248) and Beltsville (n = 505) study participants. Figure S5: Comparison of total cholesterol (mg/dL) in the two study populations. Figure S6: Comparison of HDL cholesterol (mg/dL) in the two study populations. Figure S7: Comparison of LDL cholesterol (mg/dL) in the two study populations. [file 1476-511X-12-66-S1.docx]

**Online Supporting Information**

“Single Nucleotide Polymorphisms in *CETP*, *SLC46A1*, *SLC19A1*, *CD36*, *BCMO1*, *APOA5*, and *ABCA1* are Significant Predictors of Plasma HDL in Healthy Adults.”

Authors: Andrew J. Clifford, Gonzalo Rincon, Janel E. Owens, Juan F. Medrano, Alanna J. Moshfegh, David J. Baer, and Janet A. Novotny

**Figures included:**

**Figure S1:** Diet Folate Equivalent (DFE) histogram of Sacramento (*n* = 248) and Beltsville (*n* = 505) study participants.

**Figure S2:** Red Blood Cell (RBC) folate concentrations histogram of Sacramento (*n* = 248) and Beltsville (*n* = 509) study participants.

**Figure S3**: Comparison of plasma vitamin B12 concentrations (pg/mL) in the Sacramento and Beltsville populations.

**Figure S4**: Comparison of serum triglyceride levels in the Sacramento (*n* = 248) and Beltsville (*n* = 505) study participants.

**Figure S5**: Comparison of total cholesterol (mg/dL) in the two study populations.

**Figure S6**: Comparison of HDL cholesterol (mg/dL) in the two study populations.

**Figure S7**: Comparison of LDL cholesterol (mg/dL) in the two study populations.

**Figure S1**

Comparison of dietary folate equivalent (DFE) intake assessed by the Block DFE Screener (Information available at <http://www.nutritionquest.com/assessment/list-of-questionnaires-and-screeners/>) in the Beltsville (*n* = 506) and Sacramento (*n* = 248) populations. In the Beltsville population, there were 257 female study participants (mean intake of 584 micrograms, mcg) and 253 male study participants (mean intake of 829 mcg). In the Sacramento population, there were 166 female study participants (mean intake of 826 mcg) and 82 male study participants (mean intake of 765 mcg). The bimodal distribution of the Sacramento population may reflect the large population of women of child-bearing age with high folate content in the diet.

| **DFE** | **n** | **Mean** | **Median** | **Mode** | **Std Dev** | **Variance** | **Range** |
| --- | --- | --- | --- | --- | --- | --- | --- |
| **Beltsville** | 505 | 706 | 628 | 618 | 352 | 123,919 | 2852 |
| **Sacramento** | 248 | 806 | 766 |  | 424 | 179,685 | 1806 |

**Figure S2**

Comparison of red blood cell (RBC) folate concentrations (nmol/L) in the Sacramento and Beltsville study populations that was analyzed using a previously validated LC/MS/MS method (see ref 2 of manuscript for details). The very high levels of some of the RBC folate concentrations may arise from high DFE intake (**Figure S1**) in some of the study participants. In general, however, the RBC folate concentrations agreed well between the study populations.

| **RBC Folate** | **n** | **Mean** | **Median** | **Mode** | **Std Dev** | **Variance** | **Range** |
| --- | --- | --- | --- | --- | --- | --- | --- |
| **Beltsville** | 509 | 1435 | 1198 | 854 | 819.1 | 670,932 | 6585 |
| **Sacramento** | 248 | 1203 | 1161 | 987 | 273.7 | 74,928 | 1620 |

**Figure S3**

Comparison of plasma vitamin B12 concentrations (pg/mL) in the Sacramento and Beltsville populations.

| **Plasma B12** | **n** | **Mean** | **Median** | **Mode** | **Std Dev** | **Variance** | **Range** |
| --- | --- | --- | --- | --- | --- | --- | --- |
| **Beltsville** | 506 | 542 | 505 | 441 | 244.5 | 59,757 | 1733 |
| **Sacramento** | 247 | 733 | 661 | 397 | 293.1 | 85,928 | 1876 |

**Figure S4**

The mean (± standard deviation) concentration of serum triglycerides (mg/dL) was 92.7 (57.2) for the Sacramento population and 103.4 (60.4) for the Beltsville populations. There were a few participants with very high levels of serum triglycerides, include one study participant (Sacramento) with a level of 461.0 mg/dL and another study participant with a level of 503.6 mg/dL (Beltsville).

| **TG** | **n** | **Mean** | **Median** | **Mode** | **Std Dev** | **Variance** | **Range** |
| --- | --- | --- | --- | --- | --- | --- | --- |
| **Beltsville** | 510 | 103.4 | 88.0 | 45 | 60.4 | 3645 | 484 |
| **Sacramento** | 248 | 92.7 | 77.5 | 49 | 57.2 | 3271 | 438 |

**Figure S5**

Comparison of total cholesterol values (mg/dL) in the Sacramento and Beltsville populations.

| **Total Chol** | **n** | **Mean** | **Median** | **Mode** | **Std Dev** | **Variance** | **Range** |
| --- | --- | --- | --- | --- | --- | --- | --- |
| **Beltsville** | 510 | 180.6 | 179.5 | 155.0 | 32.4 | 1045 | 203.5 |
| **Sacramento** | 248 | 200.2 | 195.0 | 210.0 | 38.2 | 1492 | 233.0 |

**Figure S6**

Comparison of HDL cholesterol values (mg/dL) in the Sacramento and Beltsville populations.

| **HDL** | **n** | **Mean** | **Median** | **Mode** | **Std Dev** | **Variance** | **Range** |
| --- | --- | --- | --- | --- | --- | --- | --- |
| **Beltsville** | 510 | 54.0 | 52.5 | 55.0 | 13.0 | 169.0 | 73.0 |
| **Sacramento** | 248 | 57.2 | 55.0 | 55.0 | 14.1 | 200.2 | 73.0 |

**Figure S7**

Comparison of LDL cholesterol values (mg/dL) in the Sacramento and Beltsville populations.

| **LDL** | **n** | **Mean** | **Median** | **Mode** | **Std Dev** | **Variance** | **Range** |
| --- | --- | --- | --- | --- | --- | --- | --- |
| **Beltsville** | 510 | 117.4 | 115.8 | 110.5 | 28.8 | 827.0 | 197.0 |
| **Sacramento** | 248 | 124.7 | 120.0 | 107.0 | 33.7 | 1134.0 | 218.0 |
